# Knockdown of miR-660 protects nucleus pulposus cells from TNF-a-induced apoptosis by targeting serum amyloid A1

**DOI:** 10.1186/s13018-019-1538-6

**Published:** 2020-01-06

**Authors:** Hao Jie Zhang, Xue Hai Ma, Song Lin Xie, Shu lian Qin, Cong Zhi Liu, Zhen Guo Zhang

**Affiliations:** 1 Department of Orthopedics, Huai An Hospital of Huai An City, No.161, Zhen Huai Lou East Road, Huai An District, Huai An City, 223200 JiangSu Province China; 2 Department of Orthopedics, Huai An Hospital of Chinese Medicine, No.3.He Ping Road, Qing Jiang Pu District, Huai An City, 223200 Jiang Su Province China

**Keywords:** MicroRNA, Apoptosis, Nucleus pulposus cell, Serum amyloid A1

## Abstract

**Background:**

Intervertebral disc degeneration (IVDD) is a well-known cause of lower back pain, which is induced by multiple factors including increased apoptosis and decreased survival of nucleus pulposus cells. In this study, we evaluate the effect and potential mechanism of miR-660 on the nucleus pulposus cells apoptosis induced by TNF-α.

**Methods:**

First, we collected tissue of nucleus pulposus from IVDD and healthy controls. General characteristic of the IVDD and healthy control was also collected. And, we also collected nucleus pulposus cells that stimulated by TNF-α or control. miRNA microarray was performed to identify the differentially expressed miRNAs. Apoptosis rate and miR-660 relative expression was measured after stimulated with different concentration of TNF-α to identify the optimal concentration of TNF-α. Second, we successfully constructed antigomiR-660 to block the miR-660 expression in nucleus pulposus cells and then stimulated with TNF-α (100 ng/ml, 12 h). The apoptosis rates and relative protein expression were then measured again. The target association between miR-660 and SAA1 was confirmed by dual-luciferase reporter.

**Results:**

There was no significant difference between the age (IVDD: 39 ± 10 years, healthy controls: 36 ± 7 years), BMI and sex between IVDD and healthy controls. Microarray analysis found that miR-660 was significantly up-regulated in IVDD and TNF-α treated groups, which was further identified by PCR. We found that the rate of apoptosis and miR-660 expression increased with TNF-α concentration increased. Finally, TNF-a with 100 ng/ml was used for further experiment. Compared with TNF-α group, TNF-α + antigomiR-660 could significantly down-regulated the apoptosis rate and relative protein (c-Caspase3 and c-Caspase7). Dual-luciferase reporter revealed that miR-660 could directly binding to the SAA1 at 80–87 sites. Compared with TNF-α alone group, TNF-α + antigomiR-660 significantly up-regulated the SAA1 expression (*P* < 0.05).

**Conclusion:**

These results indicated that knockdown of miR-660 protected the nucleus pulposus from apoptosis that induced TNF-α via up-regulation of SAA1. Further studies should focus on the role of miR-660 in protecting IVDD in vivo.

## Background

Intervertebral disc degeneration (IVDD) is a well-known cause of lower back pain and has become the most common orthopedic disease today [1, 2]. Nearly 80% of people will suffer from low back pain in their own life span [3]. Drug therapy (Non-steroidal anti-inflammatory drugs or pregabalin), vertebrectomy and decompression can alleviate the low back pain [4, 5]. However, recurrent pain and disc degeneration of adjacent segments were the weakness of these two therapies [6].

A large of studies shown that the degeneration mainly displays as a large number of the nucleus pulposus cell apoptosis and thus nucleus pulposus cells apoptosis plays a crucial role in IVDD [7, 8]. TNF-α is a key proinflammatory cytokine and could induce the apoptosis of nucleus pulposus cells [9]. Thus, TNF-α was commonly administrated as the model of IVDD [10].

MicroRNAs (miRNAs) are small single-stranded non-coding RNAs with apoptosis-promoting or anti-apoptotic roles, consisting of 20–26 nucleotides [11]. MiRNAs inhibited gene expression through binding to the 3′ untranslated regions (3’UTR) of their target mRNAs [12]. There is growing evidence that miRNAs play key roles in IVDD and miR-199a was associated with clinical grades of degeneration [13].

In this study, we performed microRNA microarray analysis for nucleus pulposus cells between the IVDD and healthy controls. We found that miR-660 was significantly up-regulated in IVDD than healthy control. Moreover, we identify the potential mechanism of miR-660 for the apoptosis of nucleus pulposus cells.

## Methods

### Sample collection

This study was approved by the Ethics Committee of Huai An Hospital of Chinese Medicine. All included patient signed and dated informed consent form. Human nucleus pulposus specimens were collected from patients with lumbar disc herniation (*n* = 3; male = 1, female = 2, age = 39 ± 10) and lumbar burst fractures as control [*n* = 3; male = 2, female = 1, age = 36 ± 7]. Human nucleus pulposus specimens were classified as grade II (lumbar burst fractures) and grade IV (IVDD patients) according to MRI [14]. Human lumbar nucleus pulposus samples were collected from patients with spine burst fractures. There was no significant difference between the IVDD and healthy controls in terms of the BMI (24 ± 2 vs 25 ± 2, *P* = 0.123). Clinical features of study population can be seen in Table 1.

**Table 1:** General characteristic of the included patients

Variable	Normal	IVDD	*P* value
Age	36 ± 7	39 ± 10	0.153
BMI	24 ± 2	25 ± 2	0.123
Sex			
Male	2	1	1
Female	1	2	

### miRNAs microarray

The isolated nucleus pulposus tissues in IVDD and healthy controls were immerse into RNA preservation solution and then stored at − 80 °C. Total RNA was extracted and RNA quality was determined by 260/280. RNA labelling and hybridization on miRNA microarray chips were performed as previously described [15]. Following background correction and data normalization with the median method, differentially expressed genes (DEGs) between IVDD and healthy controls was performed using the limma package (version 3.5.1). Volcano plot and heatmap of the differentially expressed genes (DEGs) were also obtained.

### Cell culture

Human lumbar nucleus pulposus were kept in PBS contained with 1% penicillin. Nucleus pulposus cells were isolated according to previously described method. In brief, after nucleus pulposus were cut into pieces, the pieces were then digested by 0.2% collagenase type II for 60 min. Then, cell suspension was filtered by strainer and collected the cells by centrifuge in a 1000 r and 5 min. Nucleus pulposus cells were cultured into dulbecco’s modified eagle medium (DMEM) supplemented with 10% FBS and 100 U/mL penicillin in a 37 °C, 5% CO_2_ environment.

### Cell transfection

The lentiviral vector encoding antigomiR-660 and the miR-660 vector were purchased and synthesized by Servicebio (Wuhan, Hubei Province, China). Human nucleus pulposus cells were seeded in a 24-well plate at a density of 3.0 × 10^5^ cells/well. Then, cells were randomly divided into following groups: TNF-α group, TNF-α + vector control group and TNF-α + antagomir-660 group. Following 6 h of incubation, the nucleus pulposus were collected and performed following experiments.

### Quantitative real-time RT-PCR

Total RNA was extracted by TRIzol Reagent (Invitrogen). Real-time PCR was performed according to previously described. The PrimeScript RT reagent kit (TaKaRa, Japan) was employed for complementary DNA (cDNA) synthesis, according to the manufacturer’s instructions. RT-PCR analysis was performed with the SYBR Premix Ex Taq II kit (TaKaRa) and detection on a Roche LightCycler 480 sequence detection system. U6 and GAPDH served as an internal reference for quantitation of miRNA and mRNA respectively. Primer for PCR can be seen in Table 2.

**Table 2:** Primer information of miRNA for quantitative reverse transcription

Name	Primer name	Primer sequence (5′ to 3′)
miR-660	miR-660-F	TACCCATTGCATATCGG
	miR-660-R	GTGCAGGGTCCGAGGT
s	U6-F	TGTGTCCGTCGTGGATCTGA
	U6-R	CCTGCTTCACCACCTTCTTGA

### Apoptosis assay: flow cytometry

Nucleus pulposus cells receiving different treatments were collected and washed with PBS for three times. Apoptosis was detected using an Annexin V-FITC/PI Apoptosis Kit (BD Biosciences, Franklin Lakes, NJ, USA). The collected cells were stained with annexin V-FITC and propidium iodide for 15 min according to the manufacturer’s instructions. Apoptosis was detected with a BD FACS flow cytometer (BD Biosciences).

### Western blot

Total proteins were extracted from the nucleus pulposus cells using RIPA buffer (Solarbio, Beijing, China). Protein concentration was determined by BCA Protein Assay Kit (Solarbio, Beijing, China). Equal amount of protein was resolved on a 10% SDS-PAGE gel and transferred to polyvinylidene fluoride membranes (Servicebio, Wuhan, China). The membranes were then washed with 5% nonfat milk in Tris-buffered saline plus 0.1% Tween 20. Subsequently, the membrane was incubated at 4 °C overnight with antibodies specific for c-Caspase3, c-Caspase7, MMP3, MMP13, Collagen II, Aggrecan, SSA1 and GAPDH (Santa Cruz Biotechnology, Inc., Santa Cruz, CA). After the membranes were washed three times with TBST, horseradish peroxidase-conjugated secondary antibody was incubated for 1 h at indoor temperature. The immunoreactivities were detected enhanced chemiluminescence kit (Santa Cruz Biotechnology, Dallas, TX, USA).

### Luciferase reporter constructs and assays

The 3′-untranslated region (3′-UTR) of SAA1 fragments were inserted into luciferase vector (Promega, WI, USA). NPCs were seeded in 96-well plates at 8 × 103 cells per well, and co-transfected with the vectors, miR-660 and luciferase vector. The luciferase activity was measured using a luminometer (Promega, WI, USA) after 48 h. The cells were incubated in 5% CO_2_ at 37 °C for 48 h. The dual luciferase reporter assay system (Promega Corporation) was used to measure luciferase activity. The firefly luciferase activity was normalized.

### Statistical analyses

All data are expressed as mean ± SD. Statistical analyses were performed using statistical software programs SPSS 17.0 (IBM, NY, USA) and GraphPad Prism 7 (GraphPad, CA, USA). *p* < 0.05 was considered statistically significant.

## Results

### MiR-660 was up-regulated in degenerated nucleus pulposus tissues

**Fig. 1:**
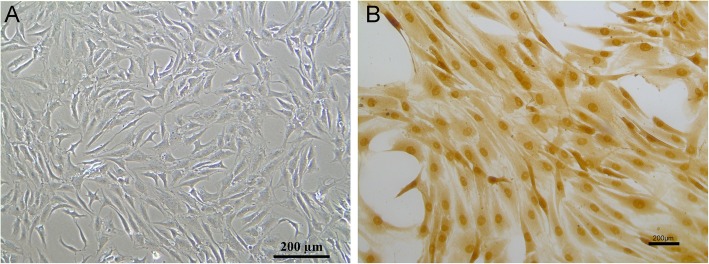
cell morphology of nucleus pulposus cells (**a**); immunohistochemical of collagen II (**b**)

**Fig. 2:**
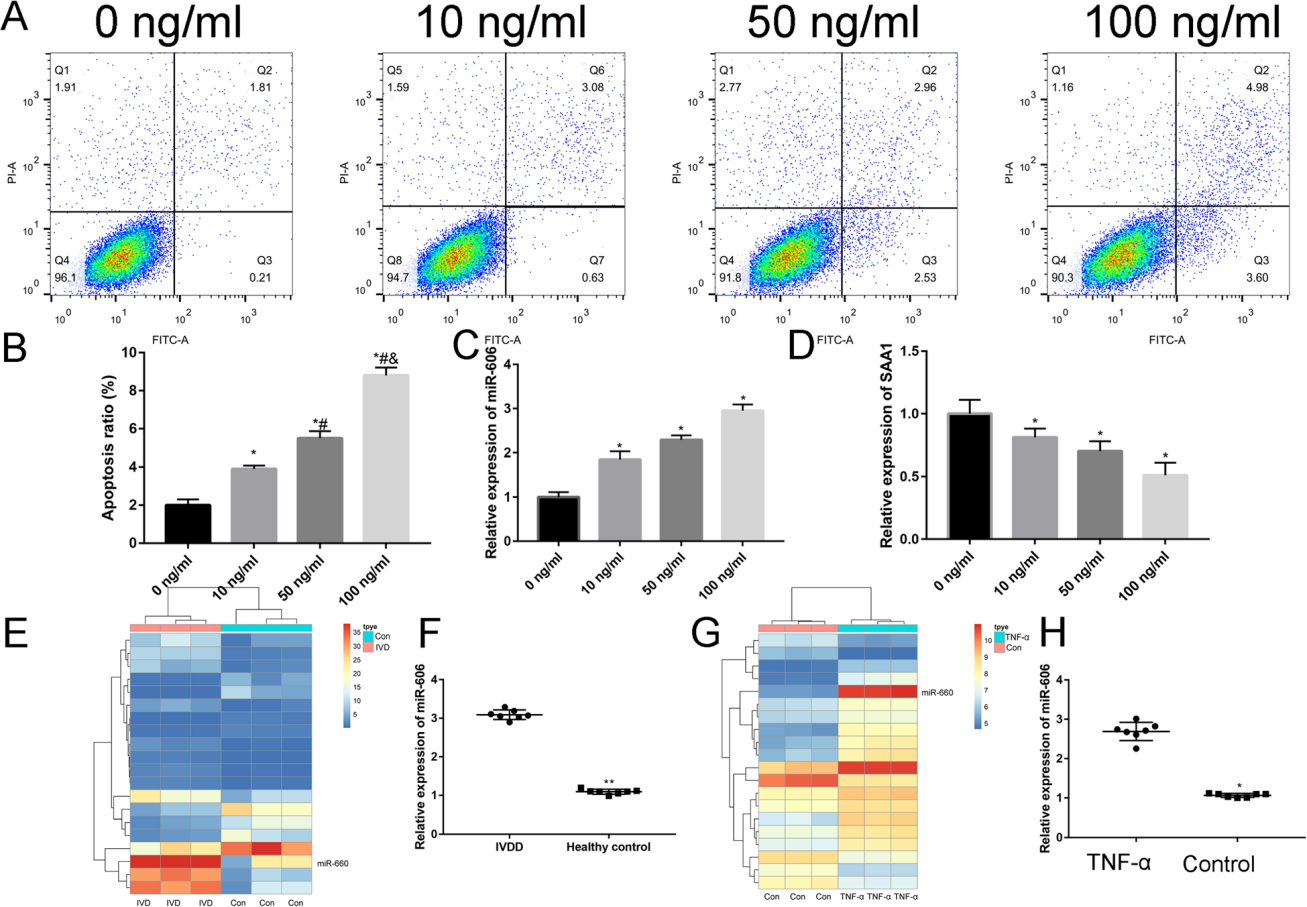
**a** Effect of TNF-α on nucleus pulposus cells apoptosis. TNF-α-induced apoptosis in concentration (0 ng/ml, 10 ng/ml, 50 ng/ml, and 100 ng/ml) at 12 h (*p < 0.05); **b** the percentages of apoptotic cells for all groups; **c** Relative expression of miR-660 in different concentration of TNF-α groups (0 ng/ml, 10 ng/ml, 50 ng/ml, and 100 ng/ml). **d** Relative expression of SAA1 in different concentration of TNF-α groups (0 ng/ml, 10 ng/ml, 50 ng/ml, and 100 ng/ml); **e** heatmap of differentially expressed genes in IVDD and healthy controls; **f** PCR result to identify the relative expression of miR-660 in IVDD and healthy control; **g** heatmap of differentially expressed genes in TNF-α and control group; **h** PCR result to identify the relative expression of miR-660 in TNF-α and control group

As shown in Fig. 1a, nucleus pulposus cells emerge as long spindle and strong expressed with type II collagen (Fig. 1b). As shown in Fig. 2a and b, TNF-α with 100 ng/ml resulted in the highest apoptosis rate than control group and other concentration of TNF-α. Thus, we administrated TNF-α (100 ng/ml, 12 h) for subsequent experiments. PCR results found that TNF-α increased miR-660 (Fig. 2c) and decreased SAA1 expression (Fig. 2d) in concentration manners.

Following gene expression data normalization, heatmap of the DEGs can be seen in Fig. 2e, and miR-660 was the most significantly up-regulated miRNA in IVD patients, which verified by PCR (Fig. 2f).

Heatmap of DEGs between TNF-α and control group can be seen in Fig. 2g, and miR-660 was the most significantly up-regulated miRNA in TNF-α treated group, which verified by PCR (Fig. 2g and h).

### MiR-660 mediates TNF-α induced nucleus pulposus cells apoptosis

**Fig. 3:**
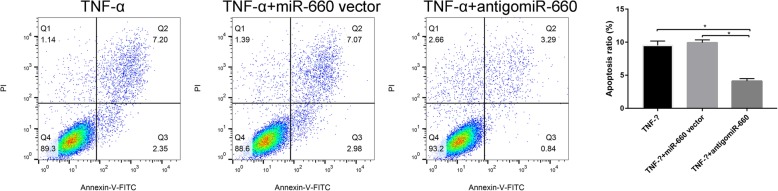
Comparison of the apoptotic cells of the different treated groups (TNF-α vs TNF-α + miR-660 vector vs TNF-α + antigomiR-660, *p < 0.05)

The rate of apoptosis was significantly decreased in the antigomiR-660+ TNF-α compared to the TNF-α alone and TNF-α + vector groups (Fig. 3, *P < 0.05). These results indicated that knockdown miR-660 could protect nucleus pulposus cells apoptosis that stimulated by TNF-α.

### MiR-660 potentially mediates cell apoptosis via targeting SAA1

**Fig. 4:**
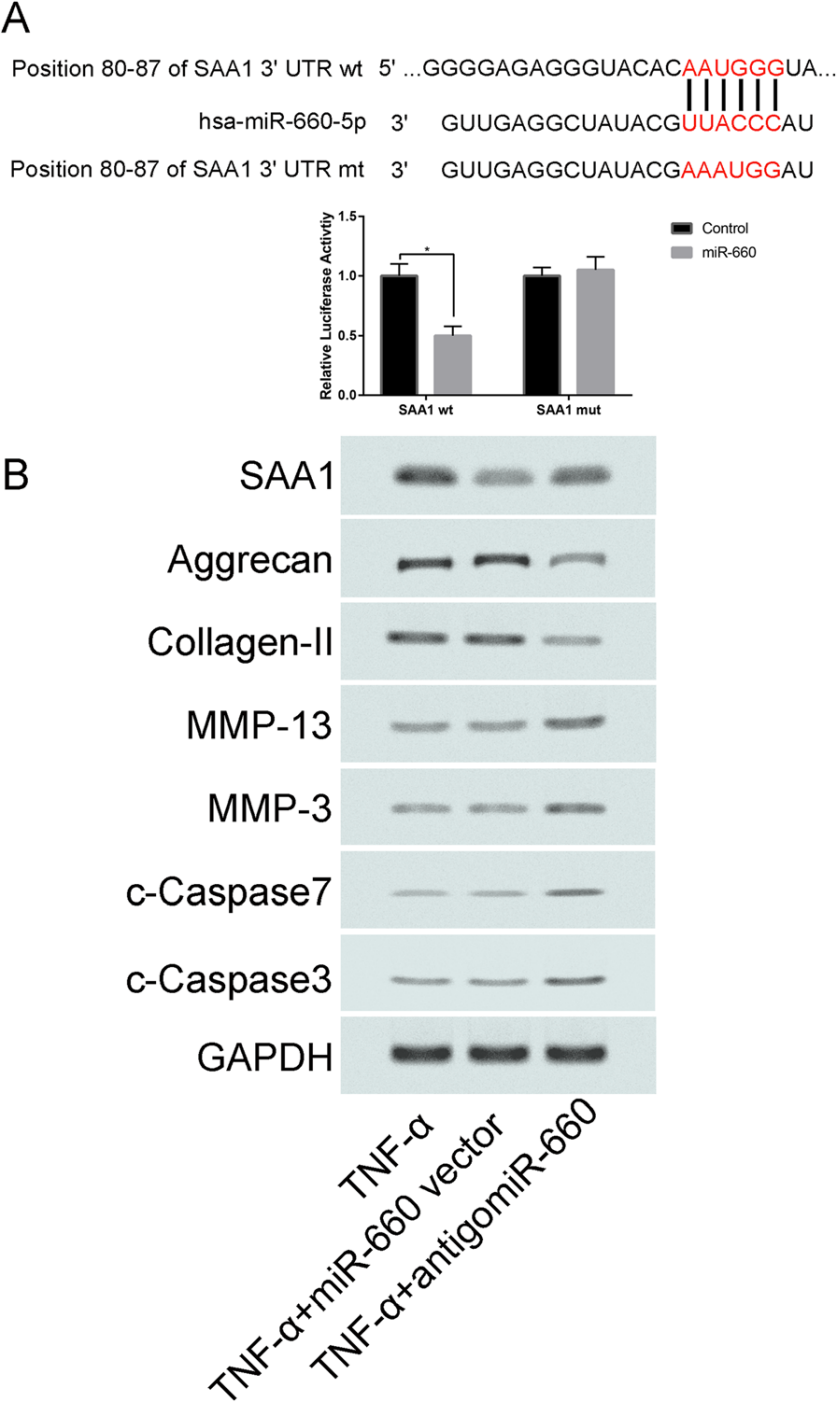
**a** schematic of SAA1 mRNA showing a potential binding site in the SAA 3′-UTR for miR-660; SAA1-mut indicates the SAA1 with the mutated sites; **b** Western-blot analyses of different protein expression (c-Caspase3, c-Caspase7, MMP-3, MMP-13, Collagen-II, Aggrecan, SAA1) in TNF-α, TNF-α + miR-660 vector and TNF-α + antigomiR-660 groups

MiR-600 had potential binding sites in the 3’UTR of SAA1 (Fig. 4a). To identify whether miR-660 can directly target SAA1 3’UTR, luciferase report vectors were generated with wild-type SAA1 3’UTR (pGL3-SAA1-WT) and mutated SAA1 3’UTR (pGL3-SAA1-MUT). The SAA1 luciferase expression vector and activity were measured for describing miR-660 function on luciferase translation. Luciferase activity of SAA1 was inhibited by miR-660 significantly, yet pGL3-SAA1-MUT relieved this effect. Then, we proved that SAA1 is the direct target of miR-660.

In addition, the expression levels of the apoptotic proteins c-Caspase3 and c-Caspase7 were significantly increased in (Fig. 4b) than TNF-α and TNF-α + miR-660 vector groups. Moreover, the expression of MMP-3, MMP-13, Collagen-II and Aggrecan in TNF-α + antigomiR-660 were significantly increased than TNF-α and TNF-α + miR-660 vector groups (Fig. 4b**,** *P* < 0.05).

## Discussion

Nucleus pulposus cells apoptosis is the main reason for IVDD [16]. In current study, we administrated with different dose of TNF-α to explore the optimal dose of TNF-α to induce apoptosis of nucleus pulposus cells. Optimal dose of TNF-α for inducing nucleus pulposus cells apoptosis was 100 ng/ml. Wang et al. [17] stimulated nucleus pulposus cells with TNF-α at different concentrations, and they found that 100 ng/ml was the optimal dose to induce apoptosis of nucleus pulposus cells. We then performed miRNAs microarray chip between nucleus pulposus cells in patient with IVDD and healthy controls. We found that miR-660 was significantly up-regulated in the IVDD patients than healthy controls. Furthermore, we identified that the relative expression of miR-660 in TNF-α treated group was higher than control group.

We found that knockdown of miR-660 protects nucleus pulposus cells from TNF-α-induced apoptosis by targeting serum amyloid A1. A major strength of current study was that we firstly identify the role of miR-660 in the process of IVDD.

In the past, some miRNAs, like miR-184 [18] and miR-640 [19], are reported to be involved in regulation of nucleus pulposus cells apoptosis. Dong et al. [19] found that miR-640 aggravates intervertebral disc degeneration via NF-κB and WNT signaling pathway. Cai et al. [20] found that miR-15a could targeting MAP 3 K9 and might be considered as a novel therapeutic target for IVDD treatment.

Firstly, we found that miR-660 was significantly up-regulated in the TNF-α treated group than control group. We used Targetscan and miRDB databases to identify the overlapping target genes of miR-660. According to the cumulative weighted context++ score, we found that SAA1 rank the first, and we further explored the role of SAA1 in the apoptosis of nucleus pulposus cells.

Serum amyloid A1 (SAA1) is a 12.5 kd acute phase protein which could induce inflammation, proliferation and cell death [21]. And previous study has shown that SAA possess an anti-apoptotic role in liver injury and other disease [22]. We found that miR-660 could directly bind to the SAA1 and thus exert an anti-apoptotic role for nucleus pulposus cells.

There are several limitations of our experiment. Firstly, although our results support that knockdown miR-660 could ameliorate nucleus pulposus cells apoptosis by regulating serum amyloid A1, the in vivo effects remain unclear. Secondly, the downstream signaling pathway was not further explored, further investigations are needed to gain deeper understanding of the role of miR-660 in IVDD.

## Conclusion

In conclusion, the present study demonstrated that knockdown miR-660 protects nucleus pulposus cells from TNF-α-induced apoptosis by targeting serum amyloid A1. These findings provide a better understanding of the mechanism involved in the pathogenesis of IVDD and miR-660 may be as a potential target for IVDD.

## Data Availability

We declare that the materials described in the manuscript will be freely available to all scientists for non-commercial purposes.

## Abbreviations

3’UTR: 3′ untranslated regions
DEGs: Differentially expressed genes
DMEM: Dulbecco’s modified eagle medium
IVDD: Intervertebral disc degeneration
miRNAs: MicroRNAs
SAA1: Serum amyloid A1
